# Tracheal stenosis in prolonged mechanically ventilated patients: prevalence, risk factors, and bronchoscopic management

**DOI:** 10.1186/s12890-022-01821-6

**Published:** 2022-01-06

**Authors:** Alessandro Ghiani, Konstantinos Tsitouras, Joanna Paderewska, Dieter Munker, Swenja Walcher, Claus Neurohr, Nikolaus Kneidinger

**Affiliations:** 1 Department of Pulmonology and Respiratory Medicine, Lung Center Stuttgart – Schillerhoehe Lung Clinic (Affiliated to the Robert-Bosch-Hospital GmbH, Stuttgart), Auerbachstrasse 110, 70376 Stuttgart, Germany; 2 Department of Internal Medicine V, Ludwig-Maximilians-University (LMU) of Munich, Munich, Germany; 3 Comprehensive Pneumology Center (CPC-M), Member of the German Center for Lung Research (DZL), Munich, Germany

**Keywords:** Mechanical ventilation, Ventilator weaning, Tracheostomy

## Abstract

**Background:**

Various complications may arise from prolonged mechanical ventilation, but the risk of tracheal stenosis occurring late after translaryngeal intubation or tracheostomy is less common. This study aimed to determine the prevalence, type, risk factors, and management of tracheal stenoses in mechanically ventilated tracheotomized patients deemed ready for decannulation following prolonged weaning.

**Methods:**

A retrospective observational study on 357 prolonged mechanically ventilated, tracheotomized patients admitted to a specialized weaning center over seven years. Flexible bronchoscopy was used to discern the type, level, and severity of tracheal stenosis in each case. We described the management of these stenoses and used a binary logistic regression analysis to determine independent risk factors for stenosis development.

**Results:**

On admission, 272 patients (76%) had percutaneous tracheostomies, and 114 patients (32%) presented mild to moderate tracheal stenosis following weaning completion, with a median tracheal cross-section reduction of 40% (IQR 25–50). The majority of stenoses (88%) were located in the upper tracheal region, most commonly resulting from localized granulation tissue formation at the site of the internal stoma (96%). The logistic regression analysis determined that obesity (OR 2.16 [95%CI 1.29–3.63], *P* < 0.01), presence of a percutaneous tracheostomy (2.02 [1.12–3.66], *P* = 0.020), and cricothyrotomy status (5.35 [1.96–14.6], *P* < 0.01) were independently related to stenoses. Interventional bronchoscopy with Nd:YAG photocoagulation was a highly effective first-line treatment, with only three patients (2.6%) ultimately referred to tracheal surgery.

**Conclusions:**

Tracheal stenosis is commonly observed among prolonged ventilated patients with tracheostomies, characterized by localized hypergranulation and mild to moderate airway obstruction, with interventional bronchoscopy providing satisfactory results.

**Supplementary Information:**

The online version contains supplementary material available at 10.1186/s12890-022-01821-6.

## Background

Intubation with mechanical ventilation may save the lives of patients with severe acute respiratory failure. However, numerous complications may arise when mechanical ventilation is prolonged, typically defined as more than 7–21 consecutive days [1]. The early risks of ventilator-associated pneumonia [2] and ventilator-induced diaphragmatic dysfunction [3] are well-known, but there are also less common late complications, such as tracheal stenosis. Several studies have been conducted on the occurrence and treatment of tracheal stenoses related to translaryngeal intubation or tracheostomy [4–8], but few have investigated patients following prolonged weaning [9].

The purpose of this study was to determine the prevalence, type, risk factors, and management of tracheal stenoses in mechanically ventilated tracheotomized patients deemed ready for decannulation following prolonged weaning.

## Methods

We conducted an exploratory, retrospective, observational study at a national weaning center. In recent years, referrals to this specialized unit have come from intensive care units across Germany. The project was approved by the local institutional review board for human studies (Ethics Committee of the State Chamber of Physicians of Baden-Wuerttemberg, Germany, file number F-2021–087) and performed following the Declaration of Helsinki. Informed consent was waived due to the retrospective nature of the analysis. The study followed STROBE guidelines for reporting observational studies [10].

### Data collection

Data were collected from the hospitals` electronic medical records and chart systems (PDMS Metavision ICU, iMDsoft, Tel Aviv, Israel; iMedOne, Telekom Healthcare Solutions, Bonn, Germany) and the bronchoscopy database (ViewPoint 6, GE Healthcare GmbH, Chalfont St. Giles, Great Britain). Baseline characteristics of patients were collected upon admission to the weaning center, including demographics, clinical features, the leading cause of intubation, and comorbidities. We describe the type, location, and severity of tracheal stenosis detected through flexible bronchoscopy. We also analyzed the features of the various tracheostomy tubes used during weaning and cannulation duration, defined as the time from tracheostomy to the diagnosis of tracheal stenosis. Moreover, interventional bronchoscopies performed on patients with tracheal stenosis were evaluated regarding their modalities, operation techniques, and outcomes.

### Patient selection

Tracheotomized patients referred to our center for weaning from prolonged mechanical ventilation [11] were screened for the analysis. We included patients in the study if they were deemed ready for decannulation after weaning completion, meeting the following requirements: severe dysphagia absent (characterized by excessive salivation with the inability to protect the airway), excessive respiratory secretions absent (generally based on suction frequency), and consciousness with preserved airway-protecting reflexes. Weaning completion may have been either successful or unsuccessful. Weaning success refers to sustained spontaneous breathing over at least seven consecutive days without signs of chronic ventilatory insufficiency (e.g., the development of hypercapnia). These patients remain ventilator-detached on discharge. Conversely, weaning failure is defined as either death during weaning or transition to domiciliary ventilation (by face mask or tracheostomy tube) due to chronic ventilatory insufficiency. Chronic ventilatory insufficiency describes recurrent hypercapnia during weaning trials (observed on at least two consecutive days), preventing the extension of spontaneous breathing, or sustained hypercapnia (on at least two consecutive occasions) occurring within seven days after weaning completion (requiring reinstitution of mechanical ventilation) [12]. Weaning trials (utilizing a T-piece) are conducted once a day, with the duration typically extended by 2–3 h per day. As such, decannulation in weaning failure patients was attempted for transition to non-invasive home ventilation. Flexible bronchoscopy is constantly performed on patients deemed ready for decannulation to rule out airway obstructions before removing the tracheostomy tube. We excluded patients who had not undergone bronchoscopy after weaning completion, died during weaning, or were discharged before the decannulation attempt.

### Diagnosis of tracheal stenosis

Protocolized liberation from the ventilator was performed as previously described [12, 13]. When patients were considered eligible for decannulation, it was always done in the bronchoscopy unit. The first step was to perform flexible bronchoscopy via the tracheal tube to determine the amount of respiratory secretions and evaluate the mid/lower trachea. Next, we inspected the upper respiratory tract, subglottic region, and upper trachea via nasal or oral access. The cannula was temporarily removed to assess the internal stoma site for injury (e.g., tracheal cartilage ring fractures) or obstruction to the airway. In addition, the tracheal cannula insertion site was determined to be either upper tracheal or subglottic (referred to as cricothyrotomy, with the tracheostomy tube inserted through the cricoid membrane). If there was no sign of obstruction, the patient remained decannulated. In the event of considerable airway obstruction (e.g., due to granulation tissue formation), we reinserted the tracheal cannula.

### Classification of tracheal stenosis

Each case of tracheal stenosis was characterized by the obstruction's type, level, and severity as determined by flexible bronchoscopy [8].

Stenosis types include local or concentric hypergranulation (originating from the internal stoma of the anterior tracheal wall), concentric scarring (fibrosis) stenosis, and fibrous web-like stenosis (a short, segmental, circumferential tracheal stricture without cartilage involvement) [4]. Stenosis levels are classified as subglottic (just beneath the vocal cords, but within the cricoid cartilage, also referred to as laryngostenosis), upper tracheal (generally at the internal stoma site), and mid/lower tracheal. We categorized stenosis severity based on reduction of the cross-sectional area of the trachea (including only stenoses with at least 20% stenosis) and the Myer-Cotton grade for subglottic stenosis (see Additional file 1: Fig. S1) [14].

### Management of tracheal stenosis

Depending on the type and level of obstruction, an interprofessional team comprising pulmonologists and thoracic surgeons referred the patient to interventional pulmonology or tracheal surgery.

An experienced interventional pulmonologist carried out all bronchoscopies performed in the bronchoscopy unit under general anesthesia combining the rigid with the flexible bronchoscope. Various techniques were utilized for the interventional procedure, including grasping forceps resection, argon plasma coagulation (APC), and photocoagulation [7, 8]. Grasping forceps resection was performed with an alligator biopsy forceps (2.0 mm fenestrated Swing Jaw, Olympus Corporation, Tokyo, Japan). The APC equipment comprised an APC probe, an argon gas source, and a high-frequency surgical unit (APC VIO, Erbe Elektromedizin GmbH, Tübingen, Germany) [15, 16]. Photocoagulation was performed with the Nd:YAG-diode laser (neodymium-doped yttrium aluminum garnet; KLS Martin MY60, Tuttlingen, Germany) (see Additional file 1: Fig. S2). The average continuous power ranged from 15 to 25 W during APC and laser, with the inspired oxygen fraction set at 0.4 throughout each operation to prevent combustion. We performed a second flexible bronchoscopy typically three days later to evaluate the procedure's outcome.

### Statistical analysis

We summarized baseline demographics and characteristics of patients through descriptive and frequency statistics. A Chi-square test or Fisher's exact test was used to analyze differences between groups in categorical variables, as appropriate. The Kolmogorov–Smirnov normality test was conducted on continuous variables to determine homogeneity of variance. Depending on the statistical distribution of these parameters, differences were analyzed through Student’s *t* test or Mann–Whitney *U* test. We performed a binary logistic regression analysis to determine the factors associated with tracheal stenosis considering baseline demographics, clinical characteristics, the primary reason for intubation, comorbidities, the tracheostomy technique, presence of a cricothyrotomy, and tracheostomy tube characteristics (e.g., the cannula outer diameter). The multivariable models (using forward selection) included variables deemed clinically significant a priori and variables with a *P* value of less than 0.2 in the bivariate analysis. Hosmer & Lemeshow and Nagelkerke R^2^ were used to evaluate the model's goodness-of-fit. Probability ratios are reported with 95% confidence intervals (95% CI). Since this was an exploratory study, no sample size was calculated, and we recruited patients to the maximum extent possible. We performed two-tailed tests; statistical significance was indicated by *P* < 0.05. The analyses were conducted with MedCalc version 19.2.5. (MedCalc Software Ltd, Ostend, Belgium).

## Results

**Fig. 1:**
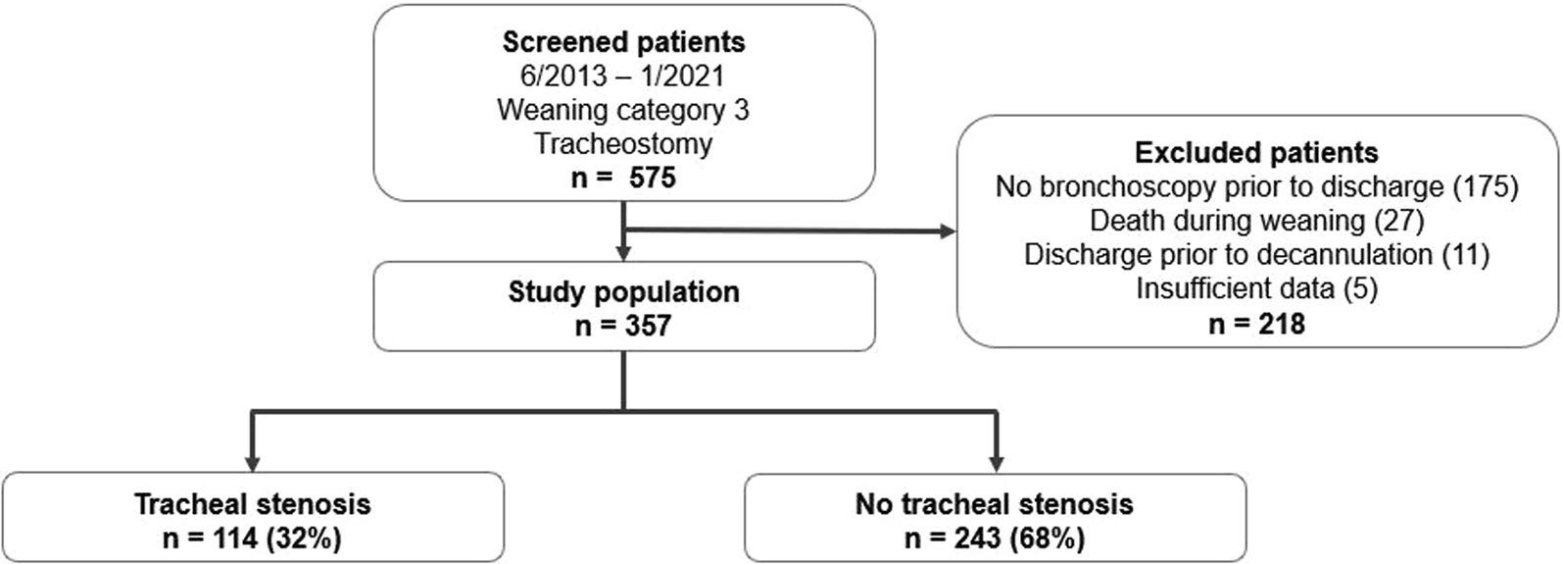
Patient flow diagram

A total of 357 of 575 (62.1%) consecutively screened patients from June 2013 to January 2021 were included in the analysis. The cases excluded were those whose upper airway bronchoscopy was lacking in 175 patients (they were not eligible for decannulation), who had died during weaning in 27, had been discharged before the first decannulation attempt in 11, and had insufficient data in five patients (Fig. 1).

On admission to the weaning center, most patients (76.2%) had percutaneous tracheostomies, and these patients were characterized by significantly reduced body mass indexes (24.8 kg/m^2^ [IQR 22.9–29.4] vs. 27.3 kg/m^2^ [24.2–32.6], *P* < 0.001). Patients with and without tracheal stenosis had similar clinical features, with only minor differences in body mass index, incidence of obesity, and serum albumin levels. Observed median times from intubation to tracheostomy were 10 days [6–14] and 36 days [27–49] from tracheostomy to the primary diagnosis of tracheal stenosis, respectively (Table 1).

**Table 1:** Clinical characteristics on admission to the weaning center—comparison of patients with and without tracheal stenosis

Clinical characteristics	All patients (n = 357)	Tracheal stenosis (n = 114)	No tracheal stenosis (n = 243)	*P* value^a^
Age (years)	69 (60–75)	69 (61–75)	69 (59–76)	0.415^b^
Male gender	216 (60.5)	66 (57.9)	150 (61.7)	0.490^c^
Body mass index (kg/m^2^)	26.2 (± 9.1)	27.3 (± 9.3)	25.7 (± 9.0)	**0.021**^**b**^
Obesity (defined as BMI ≥ 30 kg/m^2^)	93 (26.1)	40 (35.1)	53 (21.8)	**0.009**^**c**^
Smoking history	172 (48.2)	54 (47.4)	118 (48.6)	0.834^c^
APACHE-II (points)	16 (13–19)	15 (13–19)	16 (13–20)	0.449^b^
Albumin (g/dL)	2.2 (± 0.5)	2.3 (± 0.5)	2.2 (± 0.5)	**0.048**^**b**^
Pre-existing HMV-NIV	17 (6.0)	5 (4.4)	12 (4.9)	0.820^c^
Ventilator days on admission	22 (15–31)	22 (15–30)	23 (15–33)	0.947^b^
Percutaneous tracheostomy	272 (76.2)	94 (82.5)	178 (73.3)	0.057^c^
Intubation to tracheostomy (days)	10 (6–14)	10 (6–14)	10 (6–15)	0.910^b^
ECLA	29 (8.1)	9 (7.9)	20 (8.2)	0.914^c^
*Reason for mechanical ventilation*				
Pneumonia	118 (33.1)	37 (32.5)	81 (33.3)	0.870^c^
Surgery	84 (23.5)	22 (19.3)	62 (25.5)	0.197^c^
Acute exacerbation of COPD	38 (10.6)	13 (11.4)	25 (10.3)	0.750^c^
Sepsis (including septic shock)	35 (9.8)	12 (10.5)	23 (9.5)	0.754^c^
Cardiopulmonary resuscitation	28 (7.8)	11 (9.6)	17 (7.0)	0.385^c^
Acute heart failure	11 (3.1)	3 (2.6)	8 (3.3)	0.737^c^
Other	43 (12.0)	16 (14.0)	27 (11.1)	0.429^c^
*Comorbidities*				
Charlson comorbidity index (points)	5 (4–7)	5 (4–7)	5 (4–7)	0.997^b^
Diabetes mellitus	108 (30.3)	42 (36.8)	66 (27.2)	0.064^c^
Coronary artery disease	104 (29.1)	32 (28.1)	72 (29.6)	0.763^c^
Renal insufficiency	93 (26.1)	30 (26.3)	63 (25.9)	0.938^c^
Hemodialysis	43 (12.0)	15 (13.1)	28 (11.5)	0.432^c^
COPD	95 (26.6)	33 (28.9)	62 (25.5)	0.494^c^
Immunosuppression^§^	65 (18.2)	16 (14.0)	49 (20.2)	0.162^c^
Steroids*	36 (10.1)	8 (7.0)	28 (11.5)	0.188^c^
Chronic heart failure	59 (16.5)	15 (13.2)	44 (18.1)	0.241^c^
Malignancy	37 (10.4)	9 (7.9)	28 (11.5)	0.295^c^
Hepatopathy	24 (6.7)	6 (5.3)	18 (7.4)	0.451^c^
Interstitial lung disease	25 (7.0)	4 (3.5)	21 (8.6)	0.077^c^
Neuromuscular disease	4 (1.1)	0 (0.0)	4 (1.6)	0.169^c^

The bold values represent statistically significant results (with *P* < 0.05)

Continuous variables are presented as mean (± standard deviation) or median (– interquartile range [IQR]); categorical variables are presented as numbers (%)

BMI, body mass index; APACHE-II, Acute Physiology and Chronic Health Evaluation II score; HMV-NIV, home mechanical ventilation-non-invasive ventilation; ECLA, extracorporeal lung assistance (in acute respiratory failure); COPD, chronic obstructive pulmonary disease

^*^Refers to more than 20 mg of prednisolone for more than 2 weeks throughout weaning

^§^These include 36 patients on steroids, 21 patients with chemotherapy within the last three months, six after organ transplant, one with HIV stage C, and one with splenectomy

^a^*P* value for differences between patients with and without subglottic tracheal stenosis

^b^Mann-Whitney *U* test

^c^Chi-squared test

Tracheostomy tubes of different types were used before and during the weaning process, including flexible and rigid cannulas (see Additional file 1: Fig. S3). Comparing characteristics of tracheal cannulas between the groups, neither the median outer diameter (12.3 mm [10.3–12.3] for both groups during weaning, *P* = 0.429) nor cannulation duration (37 days [27–50] vs. 35 days [27–49], *P* = 0.599) before the diagnosis of tracheal stenosis differed.

There were 114 patients (31.9%) with post-tracheostomy tracheal stenosis, 109 of whom had bulky granulation tissue formation (located in the anterior tracheal wall at the site of the internal stoma), while five patients had concentric stenosis (including three patients suffering from subglottic stenosis). They showed a median percentage area stenosis of 40% [25–50] compared with 50% [40–60] for those finally referred for interventional bronchoscopy, associated with an average Myer–Cotton score of one (58%). The majority of patients (87.7%) had stenosis in the upper trachea, though 14 patients (12.3%, including 11 patients with a history of percutaneous tracheostomies) demonstrated subglottic airway obstruction. Patients with subglottic stenosis all had cricothyrotomies (the stoma was localized between the thyroid and cricoid cartilage), with a median cross-sectional reduction of 60% [50–70]. Among the patients without stenoses, six (2.5%) had cricothyroidotomies. Twenty-four patients (21.1%) with stenoses had cartilage ring fractures of the anterior tracheal wall, of which 20 had a percutaneous tracheostomy (Table 2).

**Table 2:** Characteristics and management of tracheal stenosis

Characteristics	Patients with tracheal stenosis (n = 114)
*Type of stenosis*	
Localized granulation tissue formation	109 (95.6)
Concentric granulation tissue formation	5 (4.4)
Concentric scar stenosis	0 (0.0)
Fibrous web-like stenosis	0 (0.0)
*Site of stenosis*	
Upper tracheal stenosis	100 (87.7)
Subglottic stenosis	14 (12.3)
Mid/lower tracheal stenosis	0 (0.0)
*Severity of stenosis*	
% of lumen occlusion	
All patients	40 (25–50)
Patients referred to bronchoscopy	50 (40–60)
Patients with subglottic stenosis	60 (50–70)
Myer-Cotton Grading	
Grade 1 (< 50%)	66 (57.9)
Grade 2 (50–69%)	33 (28.9)
Grade 3 (70–99%)	13 (11.4)
Grade 4 (100%)	2 (1.8)
Tracheal cartilage ring fracture	24 (21.1)
Cannulation duration (days)	37 (27–50)
*Interventional Bronchoscopy*	
No. of patients referred to bronchoscopy	77
No. of endoscopic procedures	105
*No. of procedures per patient*	
≥ 1 procedure	77 (80.2)
≥ 2 procedures	21 (21.9)
= 3 procedures	7 (7.3)
*Type of procedure*	
Nd:YAG Laser	98 (93.3)
Argon plasma coagulation	4 (3.8)
Forceps resection	3 (2.9)
Mechanical dilatation	0 (0.0)
*Severity of stenosis*	
% of lumen occlusion post-intervention	10 (0–20)
Myer-Cotton Grading post-intervention	
Grade 1 (< 50%)	76 (98.7)
Grade 2 (50–69%)	1 (1.3)
Grade 3 (70–99%)	0 (0.0)
Grade 4 (100%)	0 (0.0)
Laryngotracheal surgery	3 (2.6)

Continuous variables are presented as median (– interquartile range [IQR]); categorical variables are presented as number or number (%)

No./no., number

There were 34 patients with median area stenosis of 20% [20–30] decannulated without prior intervention. In addition, three patients not receiving bronchoscopy had high-grade subglottic stenoses causing an 80–100% reduction in tracheal cross-section (one referred to surgery, and two discharged without decannulation). On the remaining 77 patients, 105 bronchoscopic procedures were performed, with 80% undergoing only one (Table 2). Bronchoscopic procedures most commonly included Nd:YAG photo dissection of granulation tissue (93.3%). 11 out of 14 patients with subglottic stenosis were successfully treated with bronchoscopy alone, two were immediately referred for laryngotracheal reconstruction, and one was discharged without intervention due to high ventilator dependency requiring invasive home mechanical ventilation. After bronchoscopy, tracheal stenosis was graded as one according to Myer-Cotton in 99% of patients, with median residual stenosis of 10% [0–20]. There were no mechanical dilatation procedures (with the rigid bronchoscope), tracheal stenting, or Montgomery tubes placed, and only one patient underwent tracheal surgery after bronchoscopy. Bronchoscopy-related complications (e.g., bleeding, vocal cord injury, or death) did not occur. In the tracheal stenosis group, 18 patients (15.8%) failed decannulation after weaning completion, of whom four were related to airway obstruction, with a median reduction in tracheal cross-section of 75% (range 30 to 100%) at diagnosis.

**Fig. 2:**
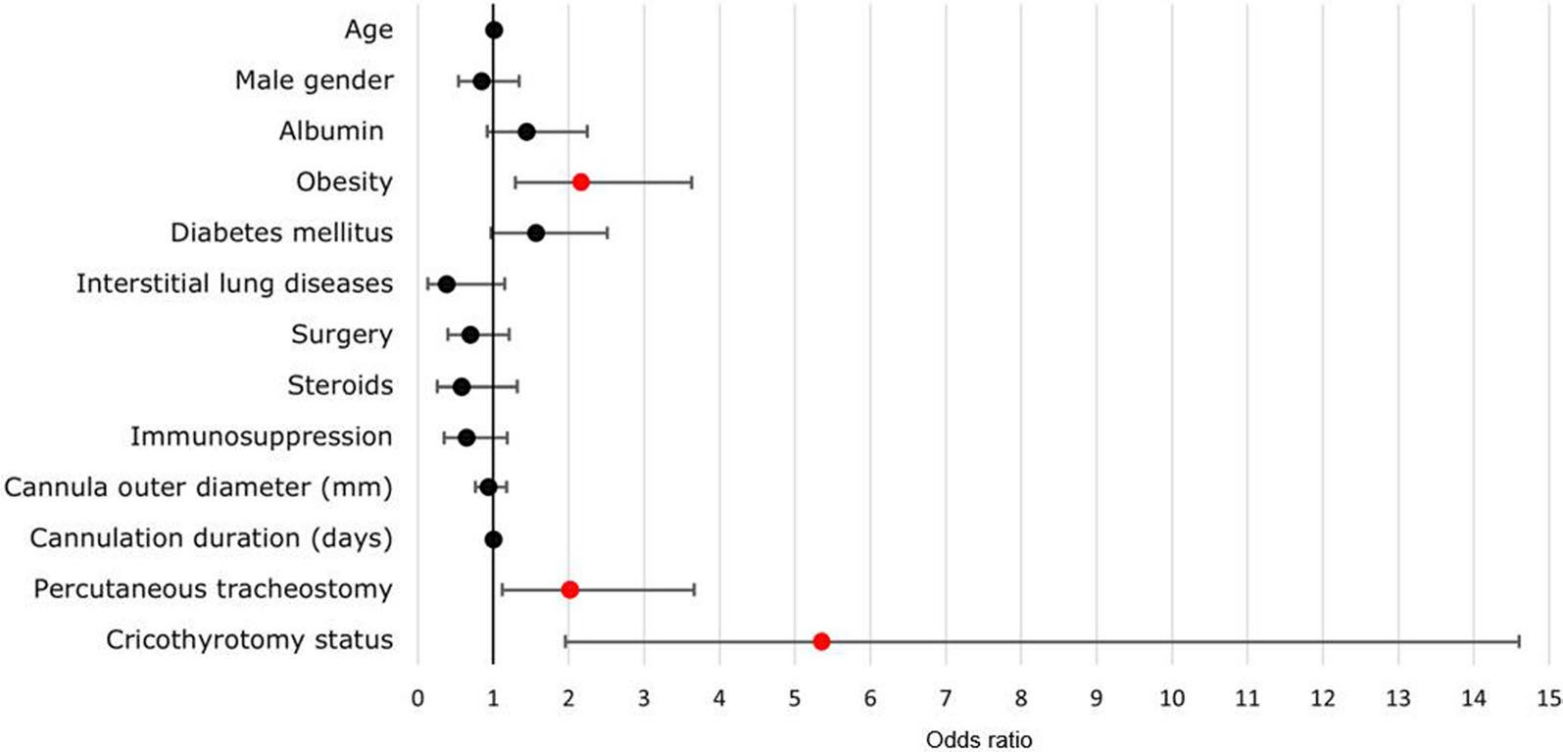
Variables associated with tracheal stenosis—results of binary logistic regression analysis. Forest plot of variables included in the univariable and multivariable (red dots) regression analysis. Odds ratios were reported with 95% confidence intervals

As determined by multivariable binary logistic regression analysis, obesity (odds ratio 2.16 [95% confidence interval 1.29–3.63]), presence of a percutaneous tracheostomy (2.02 [1.12–3.66]), and cricothyrotomy status (5.35 [1.96–14.6]) were independently related to tracheal stenosis with high specificity 98% [95% confidence interval; 95–99], poor sensitivity (12% [7–20]), and moderate accuracy (70% [65–75]) overall (Fig. 2; see Additional file 1: Tables S1 and S2).

## Discussion

This study aimed to determine the prevalence, associated risk factors, and endoscopic management of laryngotracheal stenosis following prolonged mechanical ventilation. In summary, approximately one-third of patients analyzed presented tracheal stenosis, and most of them (96%) showed bulky hypergranulation at the internal stoma, with a substantial proportion forming at the subglottic level (related to cricothyrotomy). Interventional bronchoscopy was the primary mode of treatment for most stenoses, and only three patients (2.6%) finally were referred to tracheal surgery. Among the variables included in the logistic regression analysis, obesity, percutaneous tracheostomies, and cricothyrotomies were independently related to stenosis.

Laryngotracheal stenosis from surgical and percutaneous tracheostomies is a well-known but infrequent complication [5, 17, 18] associated with unsuccessful weaning and decannulation failure in critically ill patients [9]. Since these patients have high mortality (they often die before being decannulated), and patients who survive the intensive care unit usually don't receive consistent follow-up care, it is challenging to determine the true prevalence of this condition [5, 19]. Moreover, most patients remain asymptomatic as there is typically only a subclinical airway obstruction [5, 20, 21]. Our study revealed a prevalence of 32% compared to prior reports that determined frequencies between 3.7 and 43.4% for percutaneous tracheostomies [5, 18, 21], and between 1.9 and 7.3% for surgical approaches [22, 23], respectively. Evidence suggests that tracheal stenosis is generally less common following surgical techniques [22, 24], consistent with our results. Nevertheless, some reports showed conflicting findings [19, 25], and the choice of these two techniques should not be based on the risk of tracheal stenosis [25].

With the advent of low-pressure and high-volume cuffed tubes, as well as meticulous care of the tracheostomy site, post-tracheostomy tracheal stenosis has decreased significantly [7, 26]. Several factors may predispose patients to develop stenoses, including cannulation duration [27, 28], age [21, 27], obesity [6, 7, 29], or sepsis caused by a local wound infection (surgical tracheostomy dominates) [30]. The present study revealed an independent association of tracheal stenosis with percutaneous tracheostomies and obesity. Moreover, 11 out of 14 patients (79%) with subglottic stenosis had previous percutaneous tracheostomies, likely reflecting difficulties in tracheostomy placement in general and specifically in patients with a large neck circumference [7]. Identifying additional anthropometric indicators (such as neck circumference or the chin-manubrium distance) to correlate with stenosis development would support this hypothesis. The present study also revealed an independent relationship between cricothyrotomy status and tracheal stenosis occurrence. Such stenoses have been reported in patients following translaryngeal intubation and patients who experienced damage to the circoid cartilage during percutaneous tracheostomy [30–32]. Given this, Rhaguraman and co-workers determined that patients had more stenoses closer to the vocal cords after percutaneous than surgical tracheostomy [31]. Finally, contrary to expectations, cannulation duration and the tracheal cannula outer diameter had no associations with tracheal stenosis in the present cohort.

Granulation tissue formation frequently occurred in this study, sometimes combined with tracheal cartilage ring fractures, commonly associated with tracheal stenosis following tracheostomies [7, 9, 22, 27]. By contrast, we observed no patients with web-like stenosis, which have been reported predominantly in patients after translaryngeal intubation [4].

As predicted by previous research [5, 27], stenosis was most commonly moderate, with a Myer-Cotton grading of one in about 60%, supporting the question of when to refer to bronchoscopy or tracheal reconstruction. A study by Norwood and colleagues reported that only half of the patients with mild to moderate stenosis (25–50% area stenosis) after percutaneous tracheostomy presented with respiratory symptoms. Moreover, many patients with stenosis of 75% were asymptomatic [5]. Symptoms associated with airway obstruction cannot be assessed in the present group of tracheotomized patients after prolonged weaning, but we generally refrain from immediately decannulating patients who have stenosis greater than 30%. It should be noted, however, that the evidence supporting this approach is still lacking.

The current knowledge regarding the most effective way to treat tracheal stenosis is inconclusive. However, it generally varies according to the type and extent of the disease and the expertise and experience of the center. When patients have poor performance status [4] and a high comorbidity burden, or when stenoses are web-like [6] or very lengthy, the surgical approach is generally not feasible (bronchoscopy with or without stenting is the only option). Most previous studies used the endoscopic approach in the first line of therapy combined the Nd:YAG photo dissection with or without subsequent gentle mechanical dilatation, resulting in success rates ranging between 36 and 69% [8, 9, 33]. In the present cohort, Nd:YAG photo dissection proved safe and adequate for treating stenosis, eliminating the need for further mechanical dilatation, and only a small number of patients finally required laryngotracheal reconstruction. Accordingly, most previous reports revealed that surgery was only needed in a minority of patients refractory to interventional pulmonology. Most of them exhibited so-called complex stenoses, including long segment stenoses (> 1 cm), tracheomalacia, tracheal cartilage involvement, or subglottic stenosis [4, 8, 33]. In our study, even with subglottic stenosis, photocoagulation indicated a very high success rate, which is likely due to the fact that granulation tissue formation was the primary mechanism of stenosis.

There are several strengths in our study. Considering that post-tracheostomy tracheal stenoses are late complications typically not detected during intensive care stays and that follow-up visits including bronchoscopy are uncommon, the present study provides deeper insight into prevalence and type of stenoses among prolonged ventilated patients. Further, the large sample size and the presence of a control group (without such lesions) allowed us to perform regression analyses, which revealed independent factors associated with the development of stenoses. Yet, we could also assess whether tracheostomy techniques (dilatation or surgical) were associated with stenosis.

We have, however, also identified some limitations in this study. First, external validity is uncertain due to the retrospective nature of its methodology and monocentric focus. Moreover, approximately one-third of patients screened did not qualify for decannulation after weaning, and the lack of data from this group on flexible bronchoscopy may have biased our results. Both of these factors should be considered when interpreting the study. Second, though experienced bronchoscopists assessed stenosis severity, an additional objective measurement (e.g., using CT scans) would have improved the accuracy of stenosis quantification. Third, as patients were observed only until discharge from the hospital without further follow-up, information on recurrence of stenoses is lacking. Nevertheless, recurrence rates after percutaneous tracheostomy are typically low, and most patients remain asymptomatic due to low-grade stenosis [5, 21, 34, 35]. Future prospective studies should evaluate patients with stenosis at regular intervals after discharge, including pulmonary function testing, CT imaging, and bronchoscopic airway evaluations.

## Conclusions

During this study in prolonged mechanically ventilated, tracheotomized patients, approximately one-third of patients analyzed were found to have tracheal stenosis, mainly resulting from granulation tissue formation at the internal stoma site of the upper trachea. More than half of the patients had mild to moderate stenosis, with a median reduction of 40% in the tracheal cross-section. Interventional bronchoscopy using Nd:YAG photocoagulation has proven to be a safe and highly effective first-line treatment, with only a small number of patients requiring tracheal surgery. Obesity, percutaneous tracheostomies, and cricothyrotomies were independently associated with stenoses. This study suggests that cricothyrotomies should be strictly avoided during percutaneous tracheostomy. Point-of-care airway ultrasound and bronchoscopy (e.g., identifying the cricothyroid membrane and cricoid cartilage) may help prevent tracheal stenosis.

## Supplementary Information

**Additional file 1: Fig. S1**. Myer-Cotton classification of subglottic tracheal stenosis. **Fig. S2**. Tracheal stenoses following percutaneous and surgical tracheostomies. **Fig. S3**. Different types of tracheostomy tubes used during weaning. **Table S1**. Variables associated with tracheal stenosis—results of univariable and multivariable binary logistic regression analysis. **Table S2**. Multivariable regression model.

## Data Availability

The datasets used and analyzed during the current study are available from the corresponding author on reasonable request.

## Abbreviations

95%CI: 95% Confidence interval
APACHE-II: Acute Physiology and Chronic Health Evaluation II score
APC: Argon plasma coagulation
BMI: Body mass index
COPD: Chronic obstructive pulmonary disease
ECLA: Extracorporeal lung assistance
HMV-NIV: Home mechanical ventilation‒non-invasive mechanical ventilation
IQR: Interquartile range
NdYAG: Neodymium-doped yttrium aluminum garnet
OR: Odds ratio
