# Supplementary material for: Tracheal stenosis in prolonged mechanically ventilated patients: prevalence, risk factors, and bronchoscopic management
Source: BMC Pulm Med. 2022 Jan 6;22:24. doi: 10.1186/s12890-022-01821-6 (PMC8740413; doi:10.1186/s12890-022-01821-6)
Supplement: Supplementary file 1 — Additional file 1: Fig. S1. Myer-Cotton classification of subglottic tracheal stenosis. Fig. S2. Tracheal stenoses following percutaneous and surgical tracheostomies. Fig. S3. Different types of tracheostomy tubes used during weaning. Table S1. Variables associated with tracheal stenosis—results of univariable and multivariable binary logistic regression analysis. Table S2. Multivariable regression model. [file 12890_2022_1821_MOESM1_ESM.pdf]

Tracheal stenosis in prolonged mechanically ventilated patients: prevalence, risk factors, and bronchoscopic management

Alessandro Ghiani, MD; Konstantinos Tsitouras, MD; Joanna Paderewska, MD; Dieter Munker, MD; Swenja Walcher, RT; Claus Neurohr, MD; and Nikolaus Kneidinger, MD

Additional file 1

1. Figure S1 (Page 2)
2. Figure S2 (Page 2)
3. Figure S3 (Page 3)
4. Table S1 (Page 4)
5. Table S2 (Page 4)
6. References (Page 5)

Figure S1: Myer-Cotton classification of subglottic tracheal stenoses [1]

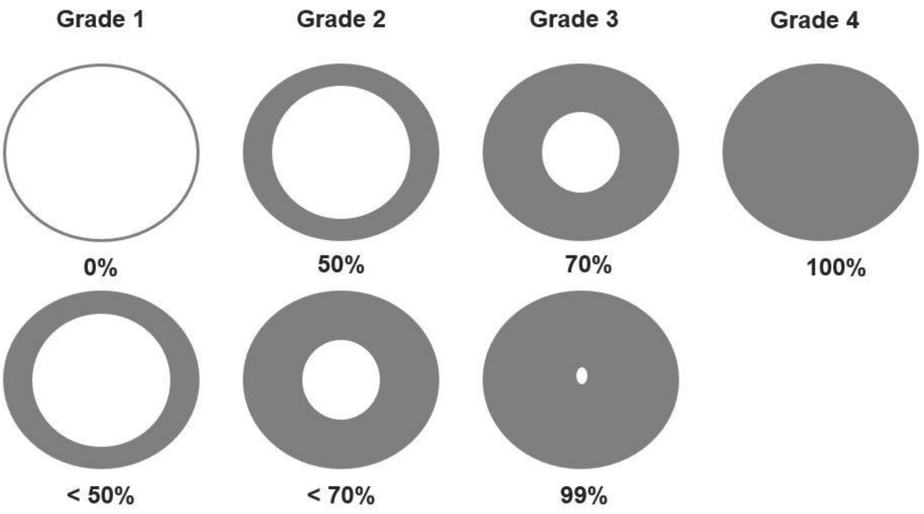

Figure S2: Tracheal stenoses following percutaneous and surgical tracheostomies

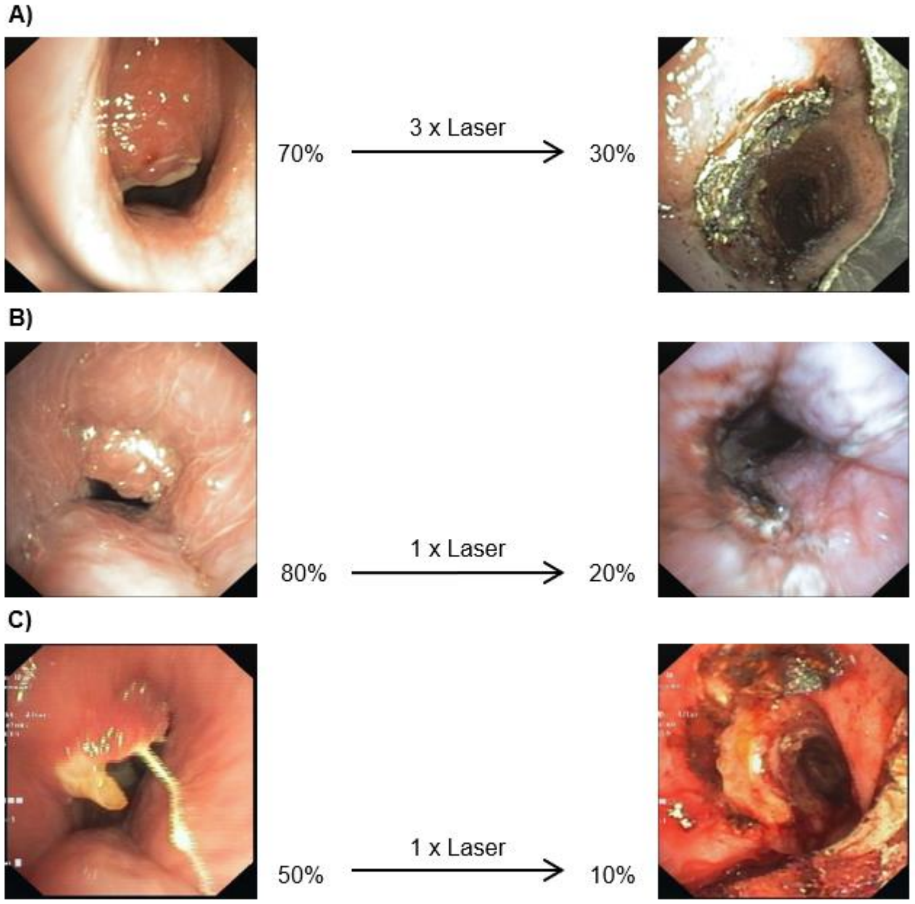

Legend
A) Subglottic stenosis (PT), B) Upper tracheal stenosis (ST), C) Upper tracheal stenosis with a tracheal cartilage ring fracture (PT); each case reported with the percentage of area stenosis before and after interventional bronchoscopy using Nd:YAG photocoagulation.
Abbreviations: PT, percutaneous tracheostomy; ST, surgical tracheostomy

Figure S3: Different types of tracheostomy tubes used during weaning

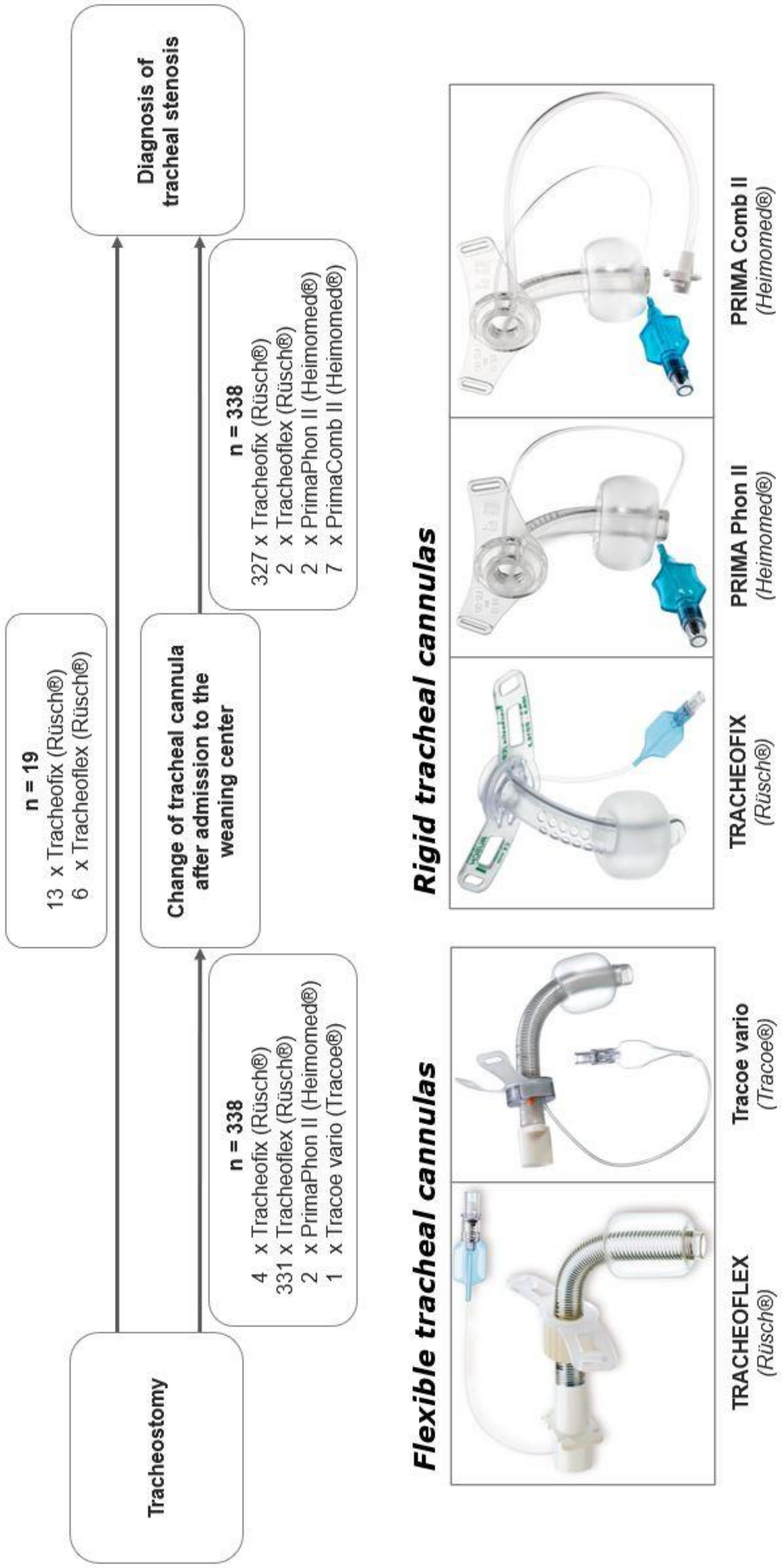

Table S1: Variables associated with tracheal stenosis – Results of univariable and multivariable binary logistic regression analysis

Variables	Univariable analysis		Multivariable analysis	
	**OR (95%CI)**	***P value***	**OR (95%CI)**	***P value***
Age	1.01 (0.99–1.03)	0.181	–	n.s.
Male gender	0.85 (0.54–1.34)	0.490	–	n.s.
Albumin	1.44 (0.92–2.25)	0.110	–	n.s.
Obesity (*defined as BMI > 30 kg/m^2*)	1.94 (1.19–3.16)	**<0.01**	2.16 (1.29–3.63)	**<0.01**
Diabetes mellitus	1.56 (0.97–2.51)	0.064	–	n.s.
Interstitial lung diseases	0.38 (0.13–1.15)	0.087	–	n.s.
Surgery	0.70 (0.40–1.21)	0.198	–	n.s.
Steroids	0.58 (0.26–1.32)	0.192	–	n.s.
Immunosuppression	0.65 (0.35–1.19)	0.164	–	n.s.
Cannula outer diameter (mm)	0.94 (0.76–1.18)	0.607	–	n.s.
Cannulation duration (days)	1.00 (0.99–1.01)	0.941	–	n.s.
Percutaneous tracheostomy	1.72 (0.98–3.00)	0.059	2.02 (1.12–3.66)	**0.020**
Cricothyrotomy status	5.53 (2.07–14.8)	**<0.01**	5.35 (1.96–14.6)	**<0.01**

Legend
The regression model variables include demographics, baseline clinical characteristics, tracheal cannula features, tracheostomy technique, and cricothyrotomy status.

Abbreviations: OR, odds ratio; 95%CI, 95% confidence interval; n.s., not significant; BMI, body mass index

Table S2: Multivariable regression model [2]

**Log-odds**	L = – 1.63 + (0.77 * obesity) + (0.70 * PT) + (1.68 * cricothyrotomy)			
**OR (95%CI); *P* value**	**obesity**	2.16 (1.29–3.63)		<0.01
	**PT**	2.02 (1.12–3.66)		0.020
	**cricothyrotomy**	5.35 (1.96–14.6)		<0.01
**Hosmer & Lemeshow**	0.902			
**Nagelkerke R^2**	0.095			
**AUROC (95%CI)**	0.63 (0.57 – 0.68)			
**2 x 2 table**	**TP** 14		**FP** 6	
	**FN** 100		**TN** 237	
**Metrics from 2 x 2 table (95%CI)**	**Sensitivity**	12% (7–20)	**PLR**	5.0
	**Specificity**	98% (95–99)	**NLR**	0.9
	**PPV**	70% (48–86)	**DOR**	6
	**NPV**	70% (69–72)	**F_1 score**	0.20
	**Accuracy**	70% (65–75)	**MCC**	0.20

Legend
Abbreviations: L, log-odds; PT, percutaneous tracheostomy; OR, odds ratio; 95%CI, 95% confidence interval; AUROC, area under the receiver operating characteristic curve; TP, true positive; FP, false positive; FN, false negative; TN, true positive; PPV, positive predictive value; NPV, negative predictive value; PLR, positive likelihood ratio; NLR, negative likelihood ratio; DOR, diagnostic odds ratio; MCC, Matthews correlations coefficient [3]

References

1. Myer CM, O`Connor DM, Cotton RT. Proposed grading system for subglottic stenosis based on endotracheal tube size. *Ann Otol Rhinol Laryngol* 1994;103: 319–323
2. Leisman DE, Harhay MO, Lederer DJ, et al.. Development and reporting of prediction models: Guidance for authors from editors of respiratory, sleep, and critical care journals. *Crit Care Med* 2020;48:623–33
3. Chicco D, Jurman G. The advantages of the Matthews correlation coefficient (MCC) over F1 score and accuracy in binary classification evaluation. *BMC Genomics* 2020;21:6
